# Porous Platinum Black-Coated Minimally Invasive Microneedles for Non-Enzymatic Continuous Glucose Monitoring in Interstitial Fluid

**DOI:** 10.3390/nano11010037

**Published:** 2020-12-25

**Authors:** Somasekhar R. Chinnadayyala, Sungbo Cho

**Affiliations:** 1 Department of Electronic Engineering, Gachon University, 1342 Seongnamdaero, Seongnam-si, Gyeonggi-do 13120, Korea; ssreddy@gachon.ac.kr; 2 Department of Health Science and Technology, GAIHST, Gachon University, Incheon 21999, Korea

**Keywords:** microneedle electrode array, electrodeposition, platinum black, glucose sensor, cyclic voltammetry, chronoamperometry

## Abstract

Individuals with diabetes can benefit considerably from continuous blood glucose monitoring. To address this challenge, a proof-of-concept was performed for continuous glucose monitoring (CGM) based on an enzymeless porous nanomaterial (pNM)-modified microneedle electrode array (MNEA). The pNM sensing layer was electrochemically deposited on MNs by applying a fixed negative current of −2.5 mA cm^˗2^ for 400 s. The pNM-modified MNEA was packed using a biocompatible Nafion ionomer. The fabricated MNEAs were 600 × 100 × 150 µm in height, width, and thickness, respectively. The surfaces of the modified MNs were characterized by scanning electron microscopy (SEM) and energy dispersive X-ray analysis (EDX), X-ray diffraction (XRD) and X-ray photoelectron spectroscopy (XPS). The fabricated MNEAs showed a wide dynamic range (1–30 mM) in phosphate-buffered saline (PBS) and in artificial interstitial fluid (ISF), with good sensitivities (PBS: 1.792 ± 0.25 µA mM^−1^ cm^−2^, ISF: 0.957 ± 0.14 µA mM^−1^ cm^−2^) and low detection limits (PBS: 7.2 µM, ISF: 22 µM). The sensor also showed high stability (loss of 3.5% at the end of 16 days), selectivity, and reproducibility (Relative standard deviations (RSD) of 1.64% and 0.70% for intra- and inter-assay, respectively) and a good response time (2 s) with great glucose recovery rates in ISF (98.7–102%).

## 1. Introduction

Due to rapid changes in blood glucose levels in patients with diabetes, frequent monitoring has become a prerequisite for strict glucose control to prevent diabetes-induced complications (e.g., heart disease, stroke, and diabetic retinopathy) [1,2]. In particular, convenient and precise glucose measuring devices yielding trustworthy data for insulin administration are needed. Conventional management of diabetes requires frequent blood sampling from the fingertips, involving discomfort, infections, and sensory loss [3]. To obviate these issues, continuous glucose monitoring systems (CGMSs) are considered a promising strategy [4]. Recently, an excellent review by Adeel et al. summarized the recent developments on the progress of non-enzymatic electrochemical, optical sensors to detect glucose in biological fluids and their potential application as wearable sensors [5]. CGMSs require a once-weekly insertion and can reduce the side effects of finger pricks, thus improving the quality of life [6]. The two main reasons for the low acceptance of CGMS by patients with diabetes are the high cost and invasiveness. Commercially available CGMSs based on retracting hypodermic needles consist of flexible sensing strips (6 mm long). These devices are inserted through the skin, which is painful and invasive [7]. Based on the needle dimensions, sensing is performed in the hypodermis, which is not an ideal site for fast and consistent glycemic monitoring [8]. In fact, interstitial fluid (ISF) is usually abundant in the dermal compartment, and the dynamics of glucose concentrations in the dermis reflect fluctuations in the blood [9]. Hence, CGMSs based on microneedles targeting ISF are gaining interest, owing to their reduced invasiveness [10].

Considering the average depth of the dermis (500–2000 µm) [11], microneedles (MNs) of <1 mm will allow direct access to the dermal ISF. Recently, microneedle electrode arrays (MNEAs) have gained significant interest in the biomedical community owing to the potential for pain-free biosensing, effective integration into biocompatible devices, and large-scale industrial production at low costs [12,13]. Advances in micro/nanofabrication have led to the clinical translation of MNEAs for the continuous monitoring of important bio-analytes in ISF (e.g., lactate, glutamate, and glucose). Recently, Goud et al., demonstrated a new electrochemical wearable microneedle sensor array for minimally invasive and continuous l-Dopa monitoring for Parkinson’s disease management [14]. The l-Dopa sensing relies on square-wave voltammetry and chronoamperometry measurements at unmodified and enzyme-modified electrodes, respectively. Windmiller et al. developed a minimally invasive carbon paste-loaded microneedle electrode array for lactate biosensing. Enzyme-loaded carbon paste microneedle electrode arrays are used for the selective detection of lactate at potential +0.15 V vs. Ag/AgCl [15]. The Wang group demonstrated a new bio-component microneedle array platform for amperometric glutamate biosensing. The microcavities of the microneedle are entrapped with the enzymes glutamate oxidase through electro-polymerization of a poly(o-phenylenediamine) thin film to prevent electro-oxidation of coexisting electroactive interferents [16]. Li et al. demonstrated a one-touch-activated blood multidiagnostic system for the detection of cholesterol. The device comprises a hollow minimally invasive biocompatible microneedle and multiplex paper-based sensor, containing the functions of blood collection, separation, and detection in one device that function by one-touch activation using finger-power [17]. Detection of glucose based on glucose oxidase MNEAs involves the reduction of hydrogen peroxide, which occurs at high (mediator-less biosensors) [18] or lower overpotentials (redox mediator-based MN biosensors) [19]. Both types of sensors showed a high interference and low stability of the mediator on the electrode surface [20]. In order to overcome these difficulties, non-enzymatic highly porous nanocomposite-based MNs facilitate a compromise in terms of higher overpotentials, sensitivity, electrode stability, and interference-free glucose detection. The highly porous platinum nanocomposite with micrometer-sized pores drastically enhanced the electroactive surface area (EASA) and the current densities when coated onto the MNEAs [21].

All commercialized CGMSs available on the market are based on electrochemical transducers, employing glucose-catalyzing enzymes as bioreceptors [22]. The advantages of electrochemical transducers include the accurate conversion of signals, simplicity, mass production, cost-effectiveness, and easy commercialization [23]. However, enzymatic CGMSs have a few critical limitations, such as instability at (a) pH values of <2 and >8, (b) in the presence of ionic surfactants, (c) at >40 °C, and (d) under oxygen limitation [24,25]. These inherent disadvantages of enzyme bioreceptors have resulted in a growing research interest in the nonenzymatic electrochemical detection of glucose.

Porous nanomaterials (pNM) are a promising alternative to enzymatic glucose monitoring systems. pNMs have recently been applied to enzymeless glucose detection in neutral PBS and ISF [26]. This approach is based on the direct electrochemical oxidation of glucose on a nanoporous surface [27]. For selective and interference-free detection, non-enzymatic highly porous nanocomposite-modified MNEAs should be packaged into bio-compatible and protective polymer films. Nafion is an attractive packaging polymer owing to its ease of handling and availability. Its fluorocarbon hydrophobic backbone and hydrophilic sulfonic acid groups form a transparent membrane with strong anion exclusion, preventing many redox-active interfering compounds from reaching the electrode array surface [28].

**Figure 1:**
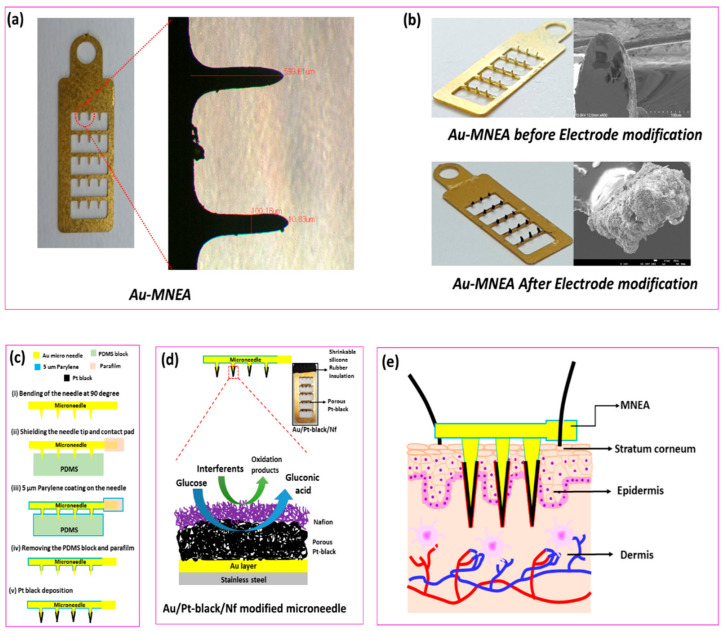
Optical micrographs of bare Au-modified microneedle electrode array (MNEA) showing a length of 599.61 µm and width of 100.16 µM (**a**). Optical and SEM Micrographs of the fabricated MNEAs before and after the catalytic Pt-black layer deposition (**b**). Schematic representation for the fabrication of MNEA (**c**) and mechanism of glucose oxidation on the surface of Pt-black modified MNEA (**d**). The schematic illustration of MNEA insertion into superficial dermis of rat skin (**e**).

In the present study, we investigated the use of a non-enzymatic porous Pt-black Au-MNEA-based sensor to determine glucose levels in ISF by a minimally invasive procedure. The porous Pt-black MN electrode was developed by electrochemical deposition through the application of a fixed negative current of −2.5 mA cm^−2^ against an Ag/AgCl external reference electrode in a solution containing chloroplatinic acid, lead acetate, and HCl. The Pt-black-modified MNs were characterized electrochemically and used for glucose monitoring in PBS and ISF. The MN electrode arrays were carefully packed in a bio-compatible Nafion membrane to prevent interferents from accessing the electrode surface. A schematic representation of the fabrication of the Au-MNEA mechanism sensor, based on the direct electrochemical oxidation of glucose on the surface of pNM and insertion of MNEA into rat skin, is presented in Figure 1.

## 2. Materials and Methods

### 2.1. Reagents and Apparatus

Refer to Supplementary Materials.

### 2.2. Fabrication of Au-MNEAs

A stainless-steel substrate (thickness: 150 µm) (316 L Grade) was patterned using a jet of wet chemical etchant, ferric chloride (FeCl3), under a 2 kgf/cm^2^ pressure for 60 s. On the patterned stainless-steel substrate, a thin gold (Au) layer was electroplated and the microneedles (MNs) were punched out of the plane (90°) using a jig. The MN tip positions and contact pads were masked using a block of PDMS and Parafilm, respectively, to prevent parylene passivation. A 5-µm-thick parylene coating was used to passivate the Au layer. After releasing the parafilm and the PDMS block, the needle tip areas were electrodeposited with the Pt-black sensing layer (Figure 1c).

### 2.3. Electrodeposition and Packaging of MNs

The Au MNs were modified with porous Pt-black using electrodeposition in a three-electrode setup (Figure S1a). The deposition was carried out in an electrolytic bath comprising 2.5% hydrogen hexachloroplatinate (IV) hexahydrate, lead diacetate trihydrate (0.05%), and HCl (0.01 M). The function of the lead diacetate trihydrate in the electrolytic bath is to decrease the over-potential, and increase the rate of Pt(IV) to Pt(0) reduction [29]. Pb ions adsorbed on the electrode surface can also act as nucleation sites for Pt islands. Pb ions decrease the conversion of Pt(IV) to Pt(II) during electrodeposition and lower the energy barrier for the reduction of Pt(IV) to Pt(0) [30]. The Pt-black electrodeposition was carried out by employing a fixed negative current of −2.5 mA cm^−2^ for 400 s vs. the Ag/AgCl external RE in a three-electrode setup. The prepared Au/Pt-black MNs were packed using a Nafion: ethanol mixture (1:6% *v/v*) by dip-coating procedure. The needles were dip-coated for 60 s in the Nafion ionomer solution and dried at 50 °C on a hot plate for 40 s followed by overnight drying at room temperature (RT) (24 °C).

### 2.4. Electrochemical Measurements

The electrochemical analyses were carried out in a two-electrode configuration (CompactStat potentiostat) consisting of a working electrode (WE) (bare and modified MNs) and a counter electrode (CE)/pseudo-reference electrode (RE) (AgCl MN). The cyclic voltammograms (CVs) were carried out in 10× PBS (pH = 7.4, 24 °C), at a sweep rate of 50 mV s^−1^ within a potential range of −0.8 V to +0.8 V vs. AgCl MN. The electrochemical impedance spectroscopy (EIS) measurements of MNs were carried out in 0.9% NaCl at room temperature (RT) (24 °C) using a three-electrode setup. The spectra were measured by selecting an alternating current perturbation voltage with a root mean square value of 0.05 V in a frequency range of 0.1 Hz–1 MHz. The Pt-black electrodeposition was carried out using a mini-potentiostat from IVIUM technologies (Eindhoven, The Netherlands) in a three-electrode configuration where, Au MN, Pt wire, and external Ag/AgCl were used as WE, CE, and RE, respectively. The amperometric measurements (I vs. t) were carried out using a two-electrode configuration in 10× PBS (pH = 7.4, 24 °C) and artificial ISF by applying an external potential of +0.12 V vs. Pt-black counter/pseudo-reference MN electrode at RT (24 °C) (Figure S1b). The electrode response was measured after background current stabilization and the analyte was spiked sequentially with increasing glucose concentrations at 50 s time intervals.

### 2.5. Preparation of ISF

The artificial ISF was formulated by adding 2.5 mM CaCl_2_, 5.5 mM glucose, 10 mM HEPES (2-[4-(2-hydroxyethyl)piperazin-1-yl]ethane sulfonic acid) (pH: 6.8), 3.5 mM KCL, 0.7 mM MgSO_4_, 123 mM NaCl, 1.5 mM NaH_2_PO_4_, and 7.4 mM saccharose. The solution pH was adjusted to 7.0 using 1 N HCl.

## 3. Results and Discussion

### 3.1. Surface Characteristics of MNEAs

#### SEM/EDX

**Figure 2:**
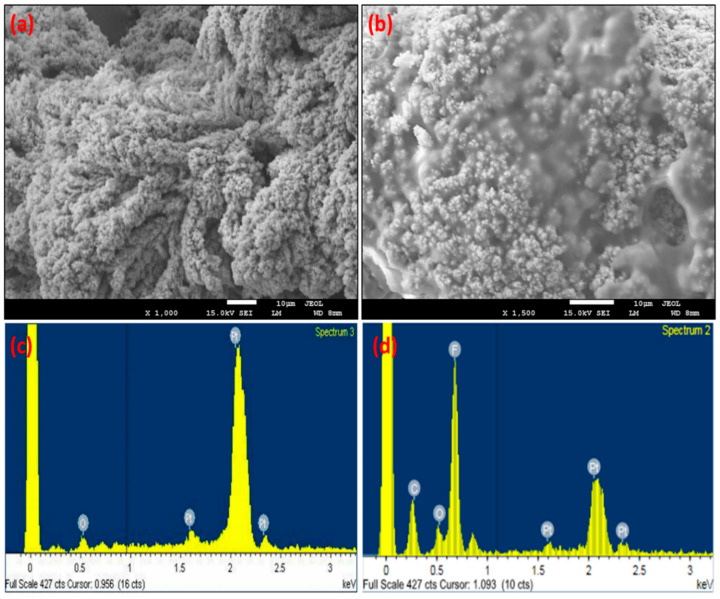
SEM images EDX spectra of Au/Pt-black MNEA (**a**) and Au/Pt-black/Nf MNEA (**b**) showing a drift change in the surface morphology after packaging the Au/Pt-black electrode with Nf ionomer. EDX spectra of Au/Pt-black MNEA (**c**) and Au/Pt-black/Nf MNEA (**d**) showing the presence of elements Pt and Pt, C, O, F for Au/Pt-black and Au/Pt-black/Nf MNEAs respectively.

The morphology of the MNEA at the major stages of electrode modification was evaluated by SEM. The micrographs show excellent needle-to-needle uniformity, as specified by the design (Figure S2a). The bare Au electrode showed a smooth surface morphology (Figure S2b). Extended complex dendritic units with porous structures were observed after Pt-black deposition on the MN tips (Figure 2a). The complex dendritic and well-defined structures transformed into porous structures after packaging with the Nafion ionomer, as shown in Figure 2b. To generate a well-defined porous structure without damaging the MN surface, a fixed negative current of −2.5 mA cm^−2^ was chosen as the optimal value for adequate hydrogen bubbling on the gold electrode surface. EDX analyses were performed to validate the elemental composition of the modified electrodes. The EDX spectrum of the Pt-black modified MNEA showed Pt (due to platinum in the metal salt) and minor amounts of O (due to atmospheric oxidation) (Figure 2c). The elemental composition of the Au/Pt-black/Nf MNEA showed high quantities of Pt stemming from the metal salt and minor amounts of C, O, and F originating from the Nafion ionomer (Figure 2d).

### 3.2. Electrochemical Characterization of the MNEAs

#### 3.2.1. EIS

**Figure 3:**
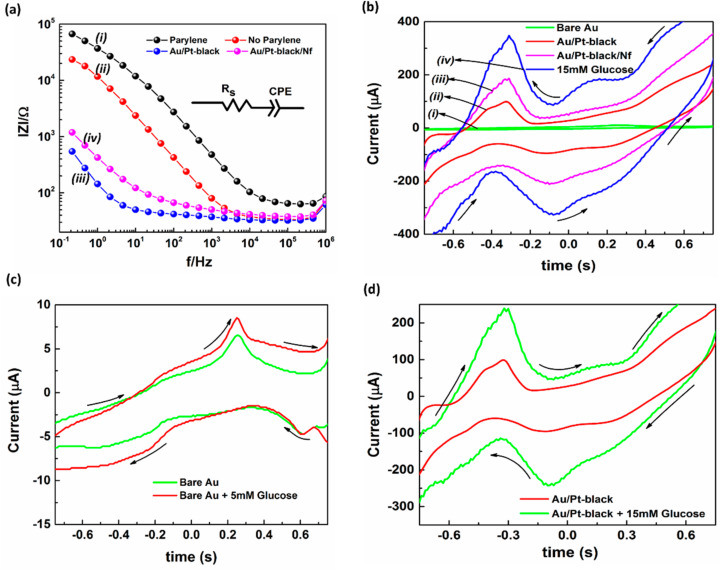
Impedance spectra of the bare Au MNs with and without parylene coating (i and ii), Au/Pt black MN (iii) and Au/Pt black/Nf MN (iv) carried out in 0.9% NaCl at a frequency range of 0.1 Hz to 1 MHz (**a**). Cyclic voltammograms (CV) of the bare Au (i), Au/Pt-black (ii), and Au/Pt-black/Nf MNs (iii) with 15 mM glucose addition (iv) at a scan rate of 50 mV·s^−1^ in 10× PBS (pH = 7.4) (**b**). The CV of the bare Au MNEA and Au/Pt-black MNEA showing the response of the electrodes with and without glucose addition (**c**,**d**).

The electrochemical impedance spectra of the MNs were measured in near neutral 0.9% NaCl (NaCl-0.9% provided the physiological conditions similar to the body fluids) by applying a range of frequency from 0.1 Hz to 1 MHz in order to probe the electrochemical characteristics of the MNEA after the Pt-black and nafion modification. The measured impedance spectra of the modified electrodes at different steps of the electrode modification were fitted to an equivalent circuit model. The electrode impedance was governed by electrode interfacial impedance modeled as the double layer capacitance (CPE) and a solution resistance in series (R_S_). The CPE is represented by T (jω)^P^, where T and P are adjustable parameters, j is the imaginary unit, and ω is the angular frequency (=2πf, f is the frequency) [31]. Table 1 summarizes the extrapolated fitting results of the measured EIS spectra for the circuit elements shown in Figure 3a. The electrodeposition of Pt-black nanomaterial led to a decrease in 1/T, suggesting an enhanced surface roughness of the overall electrode area, while P for bare MNs and modified MNs was ~1, suggesting that the interfacial impedance of electrode was mostly ascribed to the capacitive reactance. From the fitting analysis of the measured spectra in 0.9% NaCl, it was found that the impedance property of the fabricated MNs was well explained by the designed equivalent circuit model shown in Figure 3a. A bare Au MN electrode (Figure 3a, curve i) with parylene insulation and an EASA of 0.062 cm^2^ (calculated through CV in diluted sulfuric acid) [32,33] shows a very high impedance compared to the needles modified with Pt-black. The modified MNs with Pt-black (Figure 3a, curve iii) show a drastic decrease in impedance, due to a rise in the EASA of the MNs (1.520 cm^2^). The as-fabricated MNEA without parylene insulation shows a high impedance in comparison with the MNs coated with parylene (except for the MN tips) (Figure 3a, curve ii). The parylene coating aids in elimination of the direct contact between the MN and skin surface, thus completely eliminating the skin impedance. The modified MN with Pt-black and Nafion (Figure 3a, curve iv) shows a rise in impedance compared to the Au/Pt-black electrode due to the packaging of the MNs with the less conductive ionomer Nafion.

#### 3.2.2. Cyclic Voltammetry

The electrochemical characterization of the unmodified and modified MNEA was assessed by CV experiments performed in a two-electrode configuration. The CV of the bare Au MNEA showed no significant redox peaks (Figure 3b, curve i) and was electrochemically inactive in the selected potential window, whereas the Au/Pt-black electrode (Figure 3b, curve ii) showed redox peaks corresponding to the metallic Pt on the electrode in a potential ranging from −0.7 to −0.2 V vs. AgCl MN [34]. The CV of the Au/Pt-black/Nf electrode (Figure 3b, curves iii) showed redox peaks similar to those of the Au/Pt-black electrode. The CV of the Au/Pt-black/Nf electrode supplemented with 15 mM of glucose showed three pairs of peaks in the potentials ranging from −0.7 V to −0.2 V; +0.0 V to +0.3 V and +0.4 V to +0.7 V (Figure 3b, curves iv) [35]. Upon glucose addition a clear increase in current magnitude at the corresponding peak potentials was observed, suggesting the response of the modified electrode towards glucose addition (Figure 3b, curves iv). The bare Au electrodes supplemented with 15 mM glucose showed a peak at +0.28 V corresponding to the Au surface (Figure 3c) and a Au/Pt-black electrode supplemented with 5 mM glucose showed three peaks ranging from −0.7 V to −0.2 V; +0.0 V to +0.3 V and +0.4 V to +0.7 V corresponding to the glucose oxidation on the Pt surface (Figure 3d).

#### 3.2.3. Electrocatalysis of Glucose at the Modified Electrode

**Figure 4:**
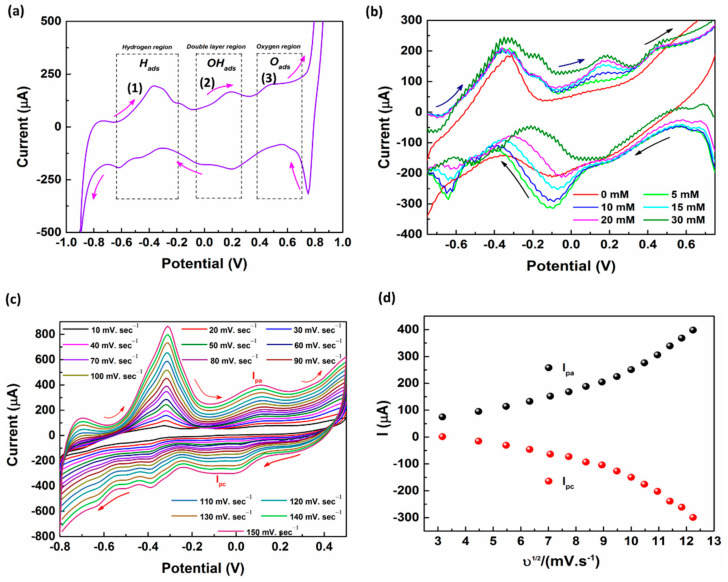
CV of the Au/Pt-black/Nf MN in 10× PBS (pH = 7.4) with 5 mM glucose addition at a scan rate of 50 mV·s^−1^ in 10× PBS (pH = 7.4) (**a**), CV of the Au/Pt-black/Nf MN in 10× PBS (pH = 7.4) with increasing concentrations of glucose between 0 and 30 mM at a sweep rate of 50 mV·s^−1^ (**b**). CVs of Au/Pt-black/Nf MNEA at various scan rates ranging from 10→150 mV s^−1^ in 10× PBS, pH 7.4 containing 5 mM of glucose (**c**), the relationship between the square root of scan rate vs. Ipa (**d**).

Upon running a cyclic voltammogram on the developed Au/Pt-black/Nf electrode supplemented with glucose (5 mM) in a potential window of −0.9 V to +0.9 V, the glucose molecules were directly oxidized on the tips of the Pt-black modified MNs into gluconic acid by the glucose adsorption followed by hydrogen removal at the C_1_ position (−0.7 V to −0.2 V). Subsequently, the water dissociates to produce hydroxide (OH) anions tailored by the oxidation of glucose (Glu_ads_) by the hydroxide anions (OH_ads_) (+0.0 V to +0.3 V). Glucose may also be oxidized by the platinum oxide layer (PtO) formed by the evolution of oxygen in electrocatalysis, thus endorsing an MN response to the glucose addition (+0.4 V to +0.7 V) (Figure 4a) [36]. The peak one represents the dehydrogenation of the glucose molecule at the hemiacetal carbon one atom (C_1_) and adsorption of the glucose molecule onto the platinum surface. The adsorption and dehydrogenation occurred at a potential region of −0.7 V to −0.2 V vs. AgCl MN reference electrode with the release of the first hydrogen atom considering as a rate-limiting step and the region is known as the “hydrogen region”. The peak two represents the electro oxidation of the chemisorbed glucose molecules which occurs in a potential range from 0.0 to +0.3 V vs. AgCl MN reference electrode, with the aid of adsorbed hydroxide anions produced by the dissociation of the water molecules. As the two electro-active species, such as hydroxyl anions and glucose molecules were adsorbed on the pt-black surface the region is called the “double-layer region”. The peak three may represent the direct catalytic oxidation of the bulk glucose solution, which occurred in a potential range from +0.4 V to +0.7 V vs. AgCl MN reference electrode. In this potential range the pt-black surface might have been covered by a monolayer of oxygen, resulting in the formation of the PtO layer. This region is called the “oxygen region” [37,38].

The voltammograms of the Au/Pt-black/Nf electrodes at various concentrations of glucose addition in 10× PBS, pH 7.4 are shown in Figure 4b. The current magnitudes at the anodic peak potential (+0.0 V to +0.3 V) of the modified MNs increased along with the step-wise increase in glucose concentration in the buffer solution from 0 mM to 30 mM. The voltage of +0.12 V vs. AgCl MN was selected as an optimum for the direct glucose oxidation in order to overcome the interference from the common interring agents in the ISF which usually occur at higher glucose oxidation over potentials (+0.4 V to +0.7 V).

#### 3.2.4. Scan Rate Study

In order to investigate the reaction kinetics, CVs of Au/Pt-black/Nf MNEAs were performed with 5 mM of glucose at different scan rates ranging from 10→150 mV.s^−1^ in 10× PBS (pH 7.4.). Figure 4c shows a shift in the oxidation peak potential (E_pa_) for the glucose oxidation peak (+0.0 → +0.3 V) towards the positive side as the scan rate increases [39], and a gradual increase in the anodic (I_pa_) and cathodic peak currents (I_pc_) with a linear increase in scan rate (ʋ) and square root of the scan rate (ʋ^1/2^). The relationship for the square root of the scan rate (ʋ^1/2^) vs. I_pa_ intensities are shown in Figure 4d. The non-linearity of (ʋ) vs. I_pa_ (Figure S3) and (ʋ^1/2^) vs. I_pa_ displays that the glucose oxidation on the modified MNEA follows a mixed diffusion kinetic controlled process, ideal for glucose oxidation (Figure 4d).

### 3.3. Effects of the Electrode Array Size and Electrodeposition Time

**Figure 5:**
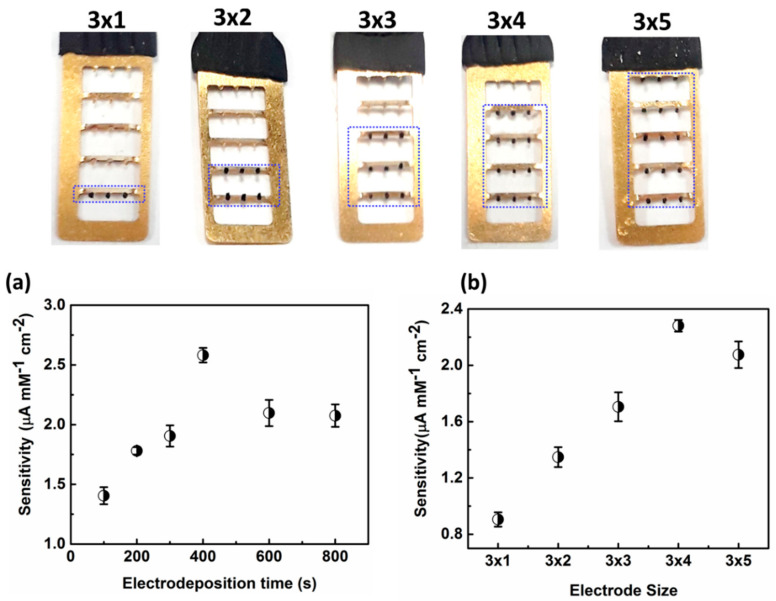
Effects of Pt-black electrodeposition time (**a**) and electrode array size (**b**) on the sensor response for continuous glucose monitoring (CGM).

To obtain the optimal electrochemical response, the electrodeposition time of pt-black (100 s, 200 s, 300 s, 400 s, 600 s, and 800 s), and electrode array size (3 × 1, 3 × 2, 3 × 3, 3 × 4, and 3 × 5 MNs) were optimized. As shown in Figure 5a, sensor activity increased as the Pt-black electrodeposition time increased until 400 s and then decreased with further increases in deposition time. Thus, the optimal electrodeposition time for the Pt-black coating on MN tips is 400 s, since higher deposition times increase the Pt-black loading on the WE, thus increasing the surface area greater than that of CE/RE. As shown in Figure 5b, sensor performance was assessed for electrode array size from 3 × 1 to 3 × 5. The sensitivity of the sensor increased as the electrode size increased from 3 × 1 to 3 × 4 and decreased above 3 × 4 due to an imbalance in the current generated during glucose oxidation between the WE and CE/RE. Hence, a 3 × 4 electrode size was used for subsequent experiments. To summarize, the optimal conditions were a Pt-black electrodeposition time of 400 s and an electrode array size of 3 × 4.

### 3.4. Response Studies of Modified MNEAs in PBS and ISF

**Figure 6:**
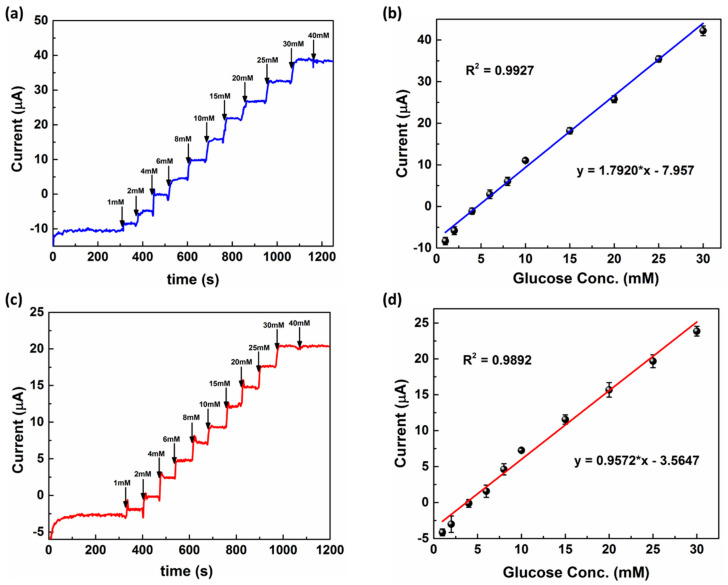
Chronoamperometric response curves (I vs. t) for the Au/Pt-black/Nf MNEAs in 10× PBS and interstitial fluid (ISF), respectively, (**a**,**c**) with successive addition of various glucose concentrations (1–30 mM) at an applied external potential of +0.12 V vs. Pt-black CE/RE. Linear calibration plots for the glucose response curves in PBS and ISF, respectively, with linear regression equations (**b**,**d**). Each data point represents the average of three independent experimental values (*n* = 3), with the range indicated in the standard error bars.

The analytical characteristics of the modified MNEAs towards the addition of glucose were assessed by I vs. t curves at an applied electrode potential of +0.12 V (Eapp = +0.12 V vs. Au/Pt-black CE/RE). Figure 6a,b show the amperometric response curves for the Au/Pt–black/Nf bioelectrode in PBS and ISF, respectively, with the successive addition of various glucose concentrations in PBS and ISF at Eapp of +0.12 V vs. Pt-black CE/RE. In both electrolytes, the modified electrodes showed a linear peak current increase at +0.12 V with increasing glucose concentrations. The MN electrodes showed a wider glucose dynamic range of 1–30 mM in both PBS and ISF (Figure 6b,d). The electrode showed a sensitivity of 1.792 ± 0.25 µA mM^−1^ cm^−2^ in PBS and 0.957 ± 0.14 µA mM^−1^ cm^−2^ in ISF, respectively. The sensitivity of the modified electrode in PBS was higher than that of the electrode in ISF due to better substrate sequestration to the catalytic layer in PBS. The higher sensitivity in turn led to a higher detection limit (DL) in the case of the modified electrode in PBS (7.2 µM) than that of the electrode in ISF (22.0 µM). The DL was calculated using the formula 3× SD/slope, where SD is the standard deviation of the bank electrode response and the slope of the calibration curves is the sensitivity of the electrode in the presence of glucose [40].

A comparative analysis on the figures of merit for various prominent minimally invasive non-enzymatic MNs reported in the past decade shows that the present Pt-black-coated minimally invasive needles offer a wide linear range of detection and an improved detection limit with acceptable sensitivity and good stability of the non-enzymatic MNs for CGM in-vitro (Table 2). The sensor showed a lower sensitivity in comparison to the studies reported in [41,42,43,44,45,46,47] but had a better dynamic range [41,42,43,44,45,46,47,48,49,50,51] and detection limit compared to the studies reported in [42,44,47,51]. The main advantages of the present study are the prevention of the parylene reactive-ion etching step which is economical for the mass production of MNEAs, the optimization of the electroplating conditions.

In order to validate the application potential of the non-enzymatic minimally invasive MN sensor, the modified Au/Pt-black/Nf electrode array was applied for the estimation of four different glucose concentrations (2.0, 7.0, 12.0, and 15.0 mM) spiked in ISF. The detailed information and the recovery rates with percentage relative standard deviation (%RSD) is given in Table S1**.** We recorded four different glucose concentration spikes at 2.0, 7.0, 12.0, and 15.0 mM, respectively, and applied the modified microneedle electrode arrays. We carried out five different measurements for each glucose concentration. The average of the five measurements (*n* = 5) was recorded and the percentage of the recovery was tabulated. It could be seen from Table S1 that the glucose concentrations in the spiked artificial ISF samples were 1.98, 6.91, 12.24, and 14.99 mM with the corresponding recoveries of 99.0% (*n* = 5), 98.7% (*n* = 5), 102.0% (*n* = 5), and 99.9% (*n* = 5), respectively. The results showed a good recovery range (98.7–102.0%; *n* = 5) for the five measurements and we found no significant difference (*p* = 0.791) between the spiked and the measured glucose concentrations using the fabricated MNEA. Hence, the Au/Pt-black/Nf modified microneedle array could be effectively used for an accurate glucose determination in ISF samples.

### 3.5. Stability, Precision, and Interference Study

**Figure 7:**
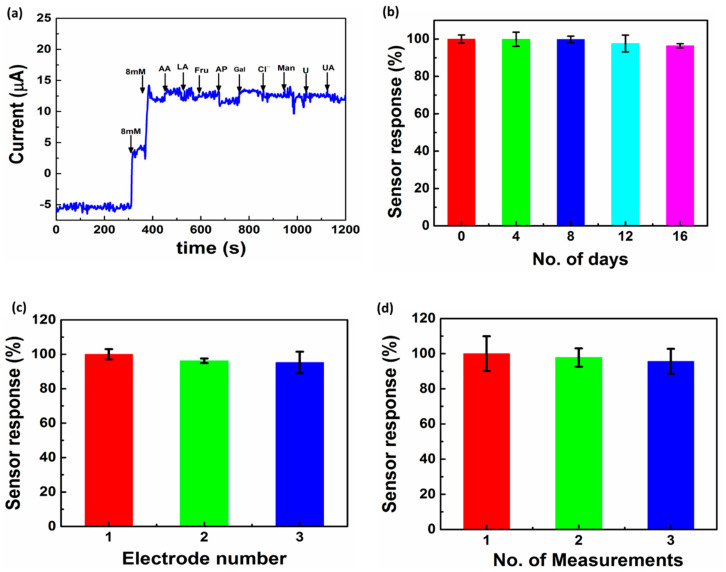
Interference study of the sensor in the presence of common interferents (AA: ascorbic acid, LA: lactic acid, Fru: fructose, AP: acetaminophen, Gal: galactose, chloride (Cl^−^), Man: mannose, urea (U) and uric acid (UA) present in ISF, carried out in 10× PBS at pH = 7.4. (**a**). Storage stability of the microneedles carried out in 10× PBS (pH = 7.4) at 24 °C (**b**), reproducibility (**c**) and repeatability (**d**) of the MNEAs studied at a concentration of 7 mM or 126.12 mg/dL of glucose, at an applied external potential of +0.12 V. Each data point represents the average of three independent experimental values (*n* = 3), with the range indicated in the standard error bars.

An important factor for the application of an MN sensor is good selectivity towards a specific analyte. To demonstrate the selectivity of the glucose MNEA sensor, the effects of certain interfering agents in ISF, i.e., ascorbic acid (0.5 mM), lactic acid (0.1 mM), mannose (0.5 mM), galactose (0.5 mM), acetaminophen (0.1 mM), fructose (0.5 mM), chloride ion from NaCl (0.5 M), uric acid (0.2 mM) and urea (0.4 mM) on the sensor response were studied in PBS (pH 7.4). The concentrations of glucose used was 7 mM (126.12 mg/dL) and the concentration of the interferents used in the investigation was 10–15-fold higher than the normal physiological level. Figure 7a shows the results obtained using Au/Pt-black/Nf microneedles in the presence of potential interferents at +0.12 V. The addition of any of these common interferents had a negligible effect on the glucose response. The limited oxidation of interferents on the electrode surface was due to the electrostatic repulsion caused by Nafion packaging on the MNEA surface [52]. Such interference-free detection reflects the strong and selective electrocatalytic activity of the Pt black MNEAs for glucose detection.

To evaluate the storage stability of the fabricated MNEAs, variation in the current response towards glucose addition (7 mM or 126.12 mg/dL) was evaluated as a function of time (Figure 7b). Over a 16-day period, the sensor response was measured at a regular interval of 4 days using the modified MNEA in 10× PBS at pH 7.4. The electrode showed a 3.5% loss in the initial response at the end of the day 16, probably due to a disturbance in the Nafion packaging. The effect of chloride ions (0.5 M NaCl) on the storage stability and the reproducibility of the sensor showed no significant effect on the sensor response (Figure S4)

Reproducibility, another key factor for the clinical use of the MNEA-based sensor, was investigated by measuring the intra- and inter-assay relative standard deviations (RSD) using a 7 mM or 126.12 mg/dL of glucose concentration. Inter-assay precision was evaluated with three different MNEAs (Figure 7c) and intra-assay precision was evaluated by three replicate measurements of a single MNEA (Figure 7d). The assays showed RSD values of 1.64% and 0.70% for intra- and inter-assay precision, respectively, demonstrating that the MNEA sensor has satisfactory repeatability and reproducibility.

## 4. Conclusions

Our results validate the application of the MN enzyme-free glucose sensor for painless and minimally invasive CGM in-vitro. The key advances of this study are the utilization of the porous Pt-black nanomaterial as the sensing layer and Nf as a packaging polymer for interference-free detection. Compared with bare Au MNs, porous Pt black increased the electroactive surface area, thus enhancing the sensitivity of the sensor. Using artificial ISF, the device exhibited a good dynamic range, DL, and recovery rate with acceptable RSDs. The device also showed good selectivity and storage stability with a 3.5% loss of the initial response after 16 days of operation in PBS. Using an animal model, the fabricated MNs were sharp enough to penetrate the superficial dermis and could easily access the ISF in the dermal compartment. These results suggest that the fabricated MNEA based on non-enzymatic sensors are promising as wearable devices for CGM in patients with diabetes. In the future, studies should focus on minimally invasive glucose sensing in live animal models and the integration of electronics for wireless communication. Furthermore, future studies should also evaluate toxicity associated with the use of the newly developed devices.

## Figures and Tables

**Table 1:** Electrochemical impedance spectroscopy (EIS) parameters of the unmodified and modified MNEAs extrapolated by fitting the measured spectra with the equivalent circuit model as shown in Figure 5a.

Electrode	R_S_ (Ω)	Constant Phase Element (CPE)		Chi Square
		T(×10^−5^ Ω^−1^·s^P^)	P	
No parylene	29.15	2.6155	0.8141	0.0534
Parylene	44.65	7.6058	0.8442	0.0743
Au/Pt-black	36.31	156.15	0.9290	0.0330
Au/Pt-black/Nf	42.52	74.244	0.8024	0.0107

**Table 2:** Comparison of the analytical figures-of-the merit for the developed Au/Pt-black/Nf MNEA sensor in PBS and ISF with other reported non-enzymatic and enzymatic sensors.

Electrode Configuration	Sensitivity (µA mM^−1^ cm^−2^)	Dynamic Range (mM)	Detection Limit (µM)	Reference
PANI@CuNi/GC	1030	0.1–5.6	0.2	[41]
Pt-Au/MWCNT	10.71	0.04–24.4	10.0	[42]
CuO NFs–ITO	873	0.2–1.3	0.04	[43]
Macroporous Au-Pt	25	1.0–20.0	25.0	[44]
Pt NTAEs	0.1	2.0–14.0	1.0	[45]
Pt_3_Ru_1_/GCE	31.3	0.005–10.0	0.3	[46]
Pt/MWNTs/graphene	11.06	1.0–7.0	387.0	[47]
PtNCs/graphene	1.21	1.0–25.0	1.0	[48]
PtNFs/GO	1.26	0.002–10.3	2.0	[49]
Pt/MWCNT	1.10	2.0–20.0	N.R	[50]
Nanoporous Pt	1.65	1.0–10.0	97.0	[51]
Au/Pt-black/Nf	1.792 ± 0.25	1.0–30.0	7.2	This work (PBS)
Au/Pt-black/Nf	0.957 ± 0.14	1.0–30.0	22.0	This work (ISF)

N.R: not reported.

## Data Availability

No new data were created or analyzed in this study. Data sharing is not applicable to this article.

## Supplementary Materials

The following are available online at https://www.mdpi.com/2079-4991/11/1/37/s1, Figure S1: Electrochemical deposition of Pt-black by potentiometry in a three-electrode configuration: using bare gold microneedle as the WE; platinum wire as the CE, and Ag/AgCl as the external RE. Electrodeposition was carried out at −2.5 mA cm^−2^ for 400 s. Figure S2: Scanning electron micrographs showing excellent needle-to-needle uniformity and spacing (a & b) with a smooth surface morphology. Figure S3: Plots of the anodic peak current (Ipa) and the cathodic peak current (Ipc) vs. the scan rate (ν). Figure S4: Effect of chloride ions on the reproducibility and storage stability of the Au/Pt-black/Nf microneedle sensor. Table S1: Recoveries and % RSDs for glucose-spiked ISF samples determined using the developed Au/Pt-black/Nf MNEAs.
