# Supplementary material for: Porous Platinum Black-Coated Minimally Invasive Microneedles for Non-Enzymatic Continuous Glucose Monitoring in Interstitial Fluid
Source: Nanomaterials (Basel). 2020 Dec 25;11(1):37. doi: 10.3390/nano11010037 (PMC7824010; doi:10.3390/nano11010037)
Supplement: Supplementary file 1 [file nanomaterials-11-00037-s001.pdf]

Supplementary material

Porous platinum black-coated minimally invasive microneedles for non-enzymatic continuous glucose monitoring in interstitial fluid

Somasekhar R. Chinnadayyala [1†] and Sungbo Cho [1,2*]

[1] Department of Electronic Engineering, Gachon University, 1342 Seongnamdaero, Seongnam-si, Gyeonggi-do, 13120, Republic of Korea; ssreddy@gachon.ac.kr

[2] Department of Health Science and Technology, GAIHST, Gachon University, Incheon, 21999, Republic of Korea; sbcho@gachon.ac.kr

* Correspondence: sbcho@gachon.ac.kr; Tel.: +82-(31)-750-5321

2.1 Chemicals and Instrumentation

Hydrogen hexachloroplatinate (IV) hexahydrate ($H_2PtCl_6.6H_2O$) (≥37.50% Pt basis), lead diacetate trihydrate (≥99%), hydrochloric acid (HCl), Nafion (5 w%, solution), d-(+)-glucose (≥99.5%), calcium chloride, 4-(2-hydroxyethyl)piperazine-1-ethane sulfonic acid (HEPES) (≥99.5%), potassium chloride, magnesium sulfate, sodium chloride, sodium dihydrogen phosphate, saccharose, ascorbic acid (98%), lactose (≥98%), d-(+)-galactose (≥99%), d-(+)-mannose (≥99%), and acetaminophen (≥99%) were purchased from Sigma Aldrich (St. Louis, MO, USA). Phosphate-buffered saline (PBS, pH = 7.4, 100 mM) and ethanol (99%) were purchased from OCI (Seoul, South Korea). All solutions were prepared using deionized water (resistivity ≥ 18 MΩ cm).

The surface morphology and elemental composition of the bare and modified MNEAs at each stage of electrode modification were analyzed using a field emission scanning electron microscope (HITACHI S-4700; Tokyo, Japan) operated at a voltage of 15 kV. The percentage and composition of each element on the modified electrode arrays were confirmed by SEM/EDX analyses. The electrochemical analysis was performed using an IVIUM CompactStat potentiostat (IVIUM Technologies, Eindhoven, Netherlands). X-ray photoelectron spectroscopy (XPS) elemental surface analysis was carried out using a PHI 5000 Versa Probe (Ulvac-PHI) spectrometer (Japan) with monochromator A1 Kα (1486.6 eV). Survey spectra were first recorded and region scans were then conducted over the C(1s), O(1S), S(2p), F(1s), Pt(4f) photoelectron binding energy regions. A 50 eV pass energy, 1 eV step size, 50 ms dwell time, and 200 μm × 200 μm X-ray spot size was used for a survey scan (range = 1200 to −5 eV). For region scans a pass energy of 20 eV, 0.1 eV step sizes, and 50 ms dwell times. All region scans were fitted using a standard Gaussian curve fit with Shirley background subtraction (Longo et al., 2015). The crystalline phase of the gold and Pt-black modified MN was characterized using a SmartLab® X-Ray diffractometer (Rigaku, Japan).

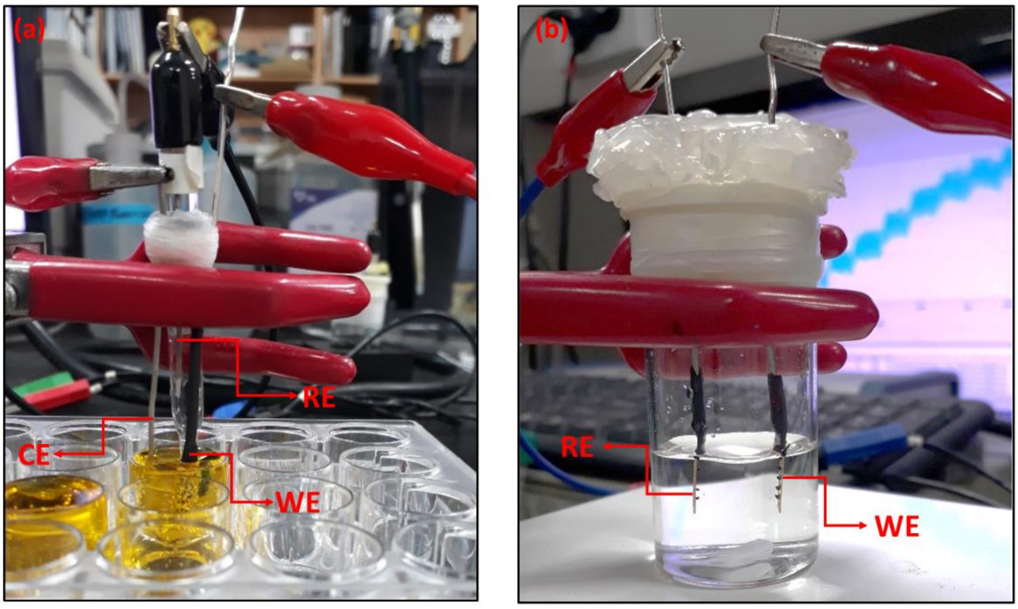

Figure. S1. Electrochemical deposition of Pt-black by potentiometry in a three-electrode configuration: using bare gold microneedle as the WE; platinum wire as the CE, and Ag/AgCl as the external RE. Electrodeposition was carried out at -2.5 mA cm^{-2} for 400 s. **(a)** Chronoamperometric experimental set-up for *in vitro* non-enzymatic glucose determination at an applied potential of +0.12 V in a two-electrode configuration using Ab/Pt-black/Nf microneedle as the working electrode (WE) and Au/Pt-black as the counter electrode/Pseudo-reference electrode (CE/RE). The electrode response was measured after the stabilization of the background currents and glucose was spiked at an interval of 50 s **(b)**.

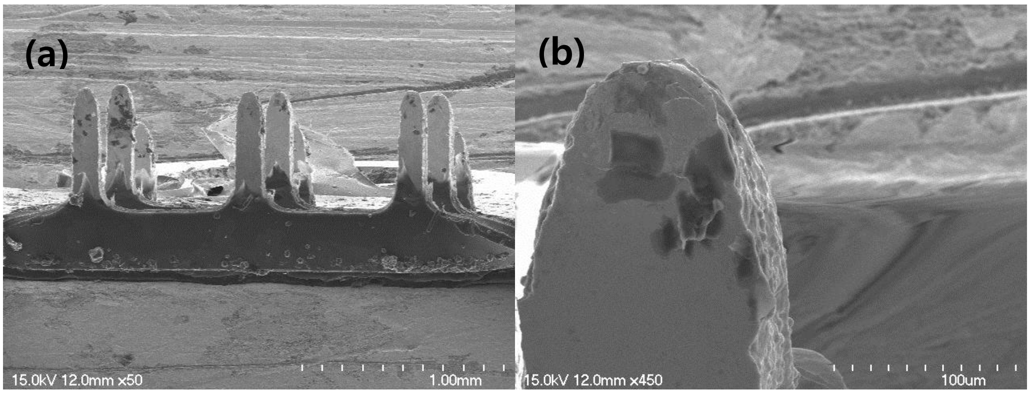

Figure. S2. Scanning electron micrographs showing excellent needle-to-needle uniformity and spacing **(a & b)** with a smooth surface morphology.

Table. S1. Recoveries and % RSDs for glucose-spiked ISF samples determined using the developed Au/Pt-black/Nf MNEAs

Test Sample	Spiked (mM)	Found (mM)	Recovery (%)	RSD (%)
A	2	1.98	99.0	1.39
B	7	6. 91	98.7	1.07
C	12	12.24	102.0	0.92
D	15	14.99	99.9	1.46

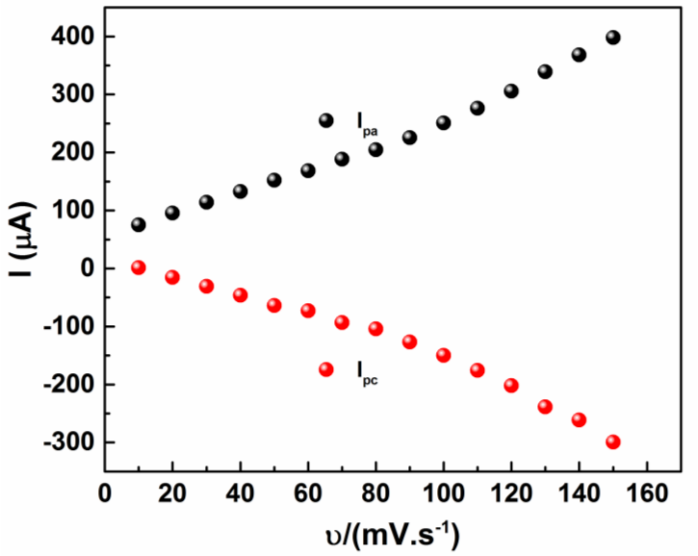

Figure. S3. Plots of the anodic peak current (Ipa) and the cathodic peak current (Ipc) vs. the scan rate (ν).

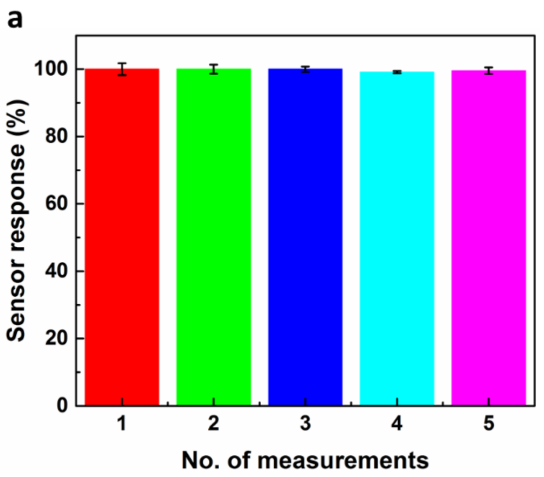

Figure. S4. Effect of chloride ions on the reproducibility and storage stability of the Au/Pt-black/Nf microneedle sensor.
